# Resilience in local Finnish health systems: how are leaders’ approaches to change manifested in organisational crisis responses?

**DOI:** 10.1108/JHOM-06-2024-0257

**Published:** 2024-12-17

**Authors:** Soila Karreinen, Kristiina Janhonen, Laura Kihlström, Henna Paananen, Marjaana Viita-aho, Liina-Kaisa Tynkkynen

**Affiliations:** Health Sciences, Faculty of Social Sciences, Tampere University, Tampere, Finland; Welfare State Research, Finnish Institute for Health and Welfare, Helsinki, Finland; Administrative Studies, Faculty of Management and Business, Tampere University, Tampere, Finland

**Keywords:** Finland, Leadership, Primary healthcare, COVID-19, Health system resilience

## Abstract

**Purpose:**

Local health systems form the basis for health system resilience. Leaders’ standpoints are crucial in advancing resilience capacities and change. This study analysed how local health system leaders’ approaches to change reflect health system resilience capacities. Furthermore, we explored what triggers and hinders change during a crisis.

**Design/methodology/approach:**

The data consist of purposively sampled interviews with 14 local Finnish health system leaders during the COVID-19 pandemic. Using abductive content analysis, examples of resisting, absorbing, adapting and transforming were identified. Contextual triggers and hindrances for the initiation of change processes were analysed to support understanding of health system resilience capacities at the local level.

**Findings:**

Resilience capacities were manifested by doing standard things faster (absorption), engaging in collaborative reflections (adaptation) and reforming organisational boundaries and services (transforming). “Resisting” leaned on varied levels of reflection, with mixed responses. Triggers and hindrances varied situationally and highlighted the roles of a changing operational environment, existing practices and the social dimension (e.g. building a shared understanding).

**Originality/value:**

Leaders’ standpoints and their approaches to change are rarely the focus of attention in system-centred conceptualisations of health system resilience. Leaders’ awareness of their approaches to change can affect organisational responses and health system resilience. This should be more clearly acknowledged in theoretical frameworks, leadership training, preparedness planning and crisis governance. Health system resilience capacities form intertwined, nonlinear processes that are reshaped throughout a crisis. Analysis of resistance can enrich the understanding of local-level processes.

## Background

Health systems are essential social structures that are increasingly subject to demands for resilience in the face of shocks and chronic stressors (Ignatowicz *et al.*, 2023; Tan *et al.*, 2023; Witter *et al.*, 2023). Local health systems have been identified as crucial yet underexplored cornerstones of health system resilience (Ignatowicz *et al.*, 2023; Saulnier *et al.*, 2023; Tan *et al.*, 2023). For example, during the acute phase of the coronavirus disease 2019 (COVID-19) pandemic, local health systems played an important role in crisis governance (Haldane *et al.*, 2021; Karreinen *et al.*, 2023a; Kihlström *et al.*, 2022), and their ability to improve organisational responses was highlighted as a crucial determinant for health system resilience (Saulnier *et al.*, 2023; Schuttner *et al.*, 2021; Seljemo *et al.*, 2023).

Aligning with these starting points, we argue that local health system leaders’ approaches to change play a pivotal role in forming resilient health systems. This is because leaders’ standpoints significantly guide the actions within their organisations as well as the organisations’ approaches to cross-sectoral collaboration (Barasa *et al.*, 2018; Karreinen *et al.*, 2023a; Ree *et al.*, 2021; Uhl-Bien and Arena, 2018). Previous research has further shown that different forms of responding to a shock require varying amounts of reflexivity in decision-making (Foroughi *et al.*, 2022; Roux-Dufort, 2000; Sarta *et al.*, 2021). Therefore, it is important to distinguish among the different types of responses and understand their determinants (Ignatowicz *et al.*, 2023).

In this article, health system resilience is regarded as the ability of the system to prepare and respond to shocks and everyday challenges (Foroughi *et al.*, 2022). We build upon the commonly used conceptualisations of resilience capacities, which include the terms absorption, adaptation and transformation (Blanchet *et al.*, 2017). These are used to describe a health system’s responses to changes in its operational environment (Biddle *et al.*, 2020; Blanchet *et al.*, 2017; Foroughi *et al.*, 2022; Thomas *et al.*, 2020). Absorptive capacity includes strategies that allow a system to continue delivering essential services by using the same level of resources and capacities, whereas adaptation underlines the need to change the modes of delivering these services to ensure properly functioning healthcare. Transforming is a health system’s ability to alter its structures and functions fundamentally in response to changes in its operational environment. (Blanchet *et al.*, 2017)

Although an increasing body of research has provided definitions of these system-level resilience capacities, there is scant research on how this plays out in practice at the local level. With this foundation, this study seeks to enrich the health system resilience literature by investigating how local health system leaders’ approaches to change link with the health system resilience capacities.

As demonstrated by previous research, shocks like the COVID-19 pandemic hold unexpected challenges within them (e.g. pandemic waves and issues of health workforce availability) that must be addressed at the local level (Karreinen *et al.*, 2023a; Seljemo *et al.*, 2023). We focused on these challenges caused by a major shock as contextual events that can either trigger or hinder the starting point of a change process. We explored how leaders’ approaches to change and the following responses to challenges can be empirically identified and classified as absorbing, adapting and transforming, which reflect resilience capacities (Blanchet *et al.*, 2017). Instead of assessing the impacts of leaders’ approaches to resilience levels achieved (see Ignatowicz *et al.*, 2023), the success of pandemic governance or drawing lessons from the COVID-19 pandemic, we focused on identifying factors that can function as triggers or hindrances to the initiation of a change process.

The following research questions guided this study:

RQ1. How do local health system leaders’ approaches to operational changes during a crisis reflect resilience capacities?

RQ2. What triggers and hinders the initiation of operational change processes during a crisis in a local health system context?

## Study design

### Collection of qualitative interview data

In Finland, many decisions regarding crisis responses, such as quarantines, school closures or health service rundowns, were made at the local level during the COVID-19 pandemic (i.e. municipalities) (Karreinen *et al.*, 2023b; Kihlström *et al.*, 2022). To achieve an accurate view of local-level operations in accordance with our research questions, we purposely sampled data from local health system leaders’ interviews from a larger dataset of 53 interviews from different levels of the Finnish health system. The 14 leaders represented four different areas in Finland, selected based on their geographical location, demography, health system organisation and epidemic situation in the spring of 2021, to provide a diverse contextual understanding of the COVID-19 pandemic response.

The participants were recruited for the interview study by using purposive and snowball sampling to represent key management positions in local health systems (i.e. municipalities, municipalities’ health and social services and joint municipal authorities) during the pandemic. The participants’ positions included city managers (*n* = 3), directors of joint municipal health and social service authorities (*n* = 2), heads of health and social services in municipalities (*n* = 5), directors of health services (*n* = 3) and an administrative head nurse (*n* = 1). All of the interviewees were members of high-level boards or groups responsible for the organisation of health and social services and crisis responses at the local level. They can be described as top- and middle-level managers in large organisations. Thus, they did not usually have a direct managerial relationship with frontline personnel. One person who was asked to be interviewed for this subsample did not respond to the invitation, while all the others agreed to participate. To ensure the respondents’ confidentiality, we do not include detailed information on the interviewees’ age, gender, positions or other descriptions combined with their regions.

The interviews were conducted by members of the research team. Online interviews were held between March and June 2021 in Finnish, each lasting 60–90 min. The participants provided verbal, audio-recorded informed consent before the interviews. The interviews were audio-recorded and transcribed verbatim. The interview guide was based on the shock cycle framework, which includes four stages of a crisis: preparedness, shock onset and alert, shock impact and management and recovery and learning (Thomas *et al.*, 2020). The interview guide had three broad domains: (1) preparedness, (2) leadership and (3) decision-making and health system resilience. The third domain included the themes of learning and adaptation. The questions focused on the processes and structures for decision-making and data gathering, supporting the workforce and ensuring adequate services.

### Abductive content analysis framework and process

After familiarising ourselves with the research literature presented above, we adopted abductive content analysis as our approach to combine different academic discussions and to connect theoretical constructs with empirical data (Timmermans and Tavory, 2012).

Our data analysis was assisted by ATLAS.ti software (Versions 9 and 23) and was conducted in four stages:

**Stage 1:** We compiled a literature-based summary of the key-term definitions: absorption, adaptation and transformation. In our definitions, we included several aspects from different researchers in addition to those of Blanchet *et al.* (2017). These are described in Table 1.
**Stage 2:** Two researchers (SK and KJ) coded the empirical manifestations of different responses to challenges brought on by the pandemic based on these preliminary definitions. Additionally, during this stage, we identified several examples of situations in which the interviewees described either personal or organisational unwillingness to act. This resistance to change can represent itself at the level of organisations, groups or individuals (Amarantou *et al.*, 2018). Many organisations are designed for stability via formal structures and hierarchical leadership cultures rather than adaptability (Uhl-Bien and Arena, 2018). These issues can further hinder both change and resilience. Consequently, we included this complementary perspective of resisting change recently brought forward in the health system resilience discourse (Topp, 2023; Foroughi *et al.*, 2022) in our coding scheme. The underlying reasons for not acting on challenges brought on by a shock are addressed later in this article. Together, the coded data extracts (identified as examples of resisting, absorbing, adapting and transforming) were interpreted in this study as leaders’ descriptions of approaches to change, according to Research Question 1. Altogether, these analyses resulted in 74 codings in the absorbing, 156 in the adapting, 31 in the transforming and 22 in the resisting categories.
**Stage 3:** We shifted our focus to a closer reading of context-bound events related to changes made during the first year of the pandemic, according to interviewees’ reflections. Here, our reading included an analysis of contextual factors that triggered or hindered the initiation of change processes (Research Question 2). These contextual triggers or hindrances were not interpreted as positive or negative *per se* concerning how they affected resilience; rather, we aimed to identify how they either facilitated or prohibited the initiation of change processes.
**Stage 4:** The definitions of our coding scheme were finalised based on our empirical notions and in accordance with the abductive analysis process. The finalised coding scheme is presented in Table 1.

**Table 1:** Definitions of approaches to change and their operationalisations used in the analysis

Approach	Definition	Operationalisation in this study
Absorbing	Strategies that aim at restoring a broader system or its activities to its original state without remarkably changing the structures of a system (Blanchet *et al.*, 2017; Foroughi *et al.*, 2022) by adding or removing something that already exists and doing familiar things at a different pace or intensity Quick responses to external stimuli, which are typically non-reflexive by nature (Roux-Dufort, 2000)	For example: shifting focus, scale or intensity of existing professional activities to ensure fulfilment of previously defined core functions of the (whole or a part of the) health system during a shock
Adapting	Strategies that aim at modifying present activities to better fit a shock at hand (Blanchet *et al.*, 2017) while aiming at restoring as much of the old procedures as possible Via adaptation, an organisation aims to change its characteristics to better match the requirements of its surroundings (Sarta *et al.*, 2021) Adaptation is more reflexive and intentional than absorption thus requiring a higher level of consciousness (Sarta *et al.*, 2021). Often, it is more structured and offers answers to more intensive challenges than does absorption (Foroughi *et al.*, 2022)	For example: modifying existing preparedness plans to better fit a challenge or rearranging existing collaborative practices to reach a more appropriate platform for information sharing for a network
Transforming	Can be characterised as fundamentally new ways of thinking or acting that produce novel types of practices (e.g. ways of acting, operational models and processes), structures or systemic components (i.e. in addition to system-wide transformations thus also including, by definition, smaller parts of a system or taking place only at a particular systemic level) (Blanchet *et al.*, 2017) Reflexively thinking about or organising an issue in a novel way, which often includes processes that force an organisation to redefine its core assumptions or value system (Roux-Dufort, 2000)	For example: redefining professional roles, expertise and skills or “high-quality care”, reorganising networking processes, interactions or the collecting of information, rethinking and reorganising multiprofessional boundaries and work practices or seeing services and their delivery more holistically
Resisting	Abstaining from action while being under pressure to change This can represent active rebellion, hesitation or stagnation (freeze mode) in the face of a challenge, and, therefore, can represent reflexive, rational, non-reflexive or emotional responses	For example: deciding not to act because of conflicting values, tensions between groups of actors, insufficient means or unclear mandates

**Source(s):** Authors' own work

### Ethical considerations

This study’s design does not require ethics approval according to the guidelines of the Finnish National Board on Research Integrity TENK (Finnish National Board on Research Integrity TENK, 2021). All the participants received written information about the study and the use of the collected data. The research conducted in this study was performed in accordance with the Declaration of Helsinki.

## Results

This section presents empirical examples of leaders’ approaches to change according to the four categories presented in the analysis section: (1) absorbing, (2) adapting, (3) transforming and (4) resisting. These are paired with our interpretations of contextual challenges identified as triggers and hindrances to the initiation of a change process.

### Absorbing: doing familiar things at a faster clock rate

Quotations reflecting an absorbing approach to change are distinguished by alterations in the pace of doing things. The interviewees typically portrayed doing “standard things at a higher clock rate”, especially during the first months of the pandemic. This would mean, for instance, daily instead of weekly board meetings, updating instructions for health professionals and the public up to several times per day, the number of nurses answering telephone calls increasing manifold and managers and leaders working long days:

I haven’t even dared to count the number of hours that I have worked in a year. As you can see, there is no resource preparedness to that extent, which means that many of us have stretched ourselves. (Interviewee 7)

The need to build a situational understanding acted as a contextual challenge that triggered vigorous information gathering at the beginning of the pandemic. In some cases, the lack of national platforms for information sharing and other technical difficulties had forced organisations to develop new means for information gathering. Thus, the contextual challenge of gathering and sharing information is a trigger for developing communication. However, technical obstacles could also function as hindrances to the building of adequate situational knowledge by making information gathering a slow, arduous task.

Existing structures of communication with other sectors and authorities, staff and the public were enhanced rapidly, providing yet another example of how the contextual challenge of an increased demand for adequate, timely information triggered the initiation of a change process:

We activated a situational awareness group that was preparing for the disturbance management group that the mayor is leading, which includes the city’s top management and rescue services. In the situational awareness group, we had people compile information on what was going on. Then we put together a pandemic group for the health and social services that has all of our leaders and managers, practically 30 people, and meets after the situational awareness group meeting. So everyone gets the information straight ahead. (Interviewee 10)

Official declarations (national and international), media and unofficial ways of obtaining information raised awareness of the crisis. This awareness, in turn, functioned as a trigger for shifting into a so-called crisis-operation mode in organisations. This exemplifies the initiation of a change process, aligning with our definition of absorption (see Table 1). These information-related challenges form a major group of triggers and hindrances in the absorbing category.

Organisations’ shift to a “crisis mode” was largely dictated by the already existing contingency plans combined with steering from higher authorities. The contingency plans, as well as other pre-defined or previously familiar ways of organising actions, guided the modification of management procedures and service delivery, allowing personnel to act fast as the shock hit the organisations.

In the later stages of the pandemic, the contextual challenge of a changing epidemiological situation was a common trigger for initiating change processes aligning with our definition of absorption (see Table 1). The significance of planning before a crisis situation was described by one interviewee as follows:

In the influenza plan, we had pre-thought in great detail all our locations with their facilities, staff and how to separate this kind of pandemic reception from other activities. Everything was ready, so it was really easy. The basic parameters and staff volumes for all of these were available. (Interviewee 5)

In line with Table 1, we interpreted the interviewees’ reflections on minor changes to organisational structures and processes as representing absorption. These included examples in which activities would be allocated or focused differently, scaled up or down (e.g. the use of digital tools) or slightly restricted (e.g. elective surgery). The initiation of these change processes was often triggered by scarce resources (human or material) both at the level of service delivery and management or the social dimension exemplified by attitudes or fears, both in professionals and the public.

In addition to attempts to prevent the spread of COVID-19, these absorptive modifications were triggered by the objective of safeguarding basic and critical services:

As far as oral health care is concerned, of course, we had to reduce the services a little bit. The backlog has been dealt with rigorously. In my opinion, basic health care could be secured pretty well after all. (Interviewee 3)

The shortage of material resources as a contextual challenge had led to a great need for the fast acquisition of personal protective equipment and other material resources, thus triggering absorbing mechanisms such as joint procurements between organisations and unusual routes for purchasing. Conversely, social factors, such as pressure from the media, conflicting interests, differing understandings of the situation and even matters of prestige, sometimes functioned as hindrances to these efforts.

### Adapting: thinking and doing based on collaborative reflections

Adapting can be described by including a more reflective approach to, for instance, modifications in services, management structures and processes (Table 1). The research data revealed that a typical contextual challenge that functioned as a trigger for such an approach was changes in the operational environment due to shifts in the epidemiological situation. Also, the interviewees often mentioned their aspiration to preserve services and ensure health security for the public and the workforce. These aspirations are here interpreted as contextual challenges of the social dimension that triggered the initiation of adaptive change processes. The adaptive processes included the use of new or upscaled digital tools or services, changing the focus or allocation and even some rundowns of health and social services.

Previous operating models in service delivery and management were identified as hindrances to adapting. Workforce reluctance (including their justifiable demands on health security measures), along with conflicting interests or differing understandings of a situation, were other examples of hindrances to adapting. These can be interpreted as representing the social dimension, which was also accentuated in descriptions of fatigue, fear, collaboration, sometimes contested distribution of power and responsibility and differing attitudes and interests. In contrast, pressure from higher administrative levels of the health system in the form of guidance and commands acted as both a trigger and hindrance, depending on situational factors (e.g. adaptive service changes could not be made if their customary delivery was obliged by guidelines or regulations). The economic hardship (i.e. limited material resources) of an organisation and information steering contradicting legislation were also mentioned by the participants and interpreted here as hindrances to initiating adaptive change processes.

Examples in the data that could be interpreted to align with the dimension of adapting (Table 1) had a certain pragmatic and agile quality. This is exemplified by quotes in which the interviewees described the contextual challenge of needing to ensure that instructions based on normative and information steering and contingency plans were realisable in practice. Accordingly, a very common example of a trigger that initiated an adaptation process concerned the modifications made to contingency or epidemic plans due to changes in an operational environment and a deeper understanding of them:

Of course, it has now been noticed that, in some respects, it [the preparedness plan] has still been on a too general or high level. You can’t make a plan for every situation, but we have now specified ours during this time. We have done action cards of one A4 sheet for different situations, where we have concretised how to act in certain situations. It’s a lesson that has been clearly learned from this. (Interviewee 1)

Collaboration in different networks was a substantial part of adapting to shocks in health service environments. This was triggered by the need to build a profound and shared understanding in an exceptional situation, to provide clear and timely communication and to ensure regionally equitable decisions on restriction measures.

Networking was mirrored in commentaries about building new collaborative groups in which leaders would strive for the coordination of local pandemic governance:

So, there are a few municipalities whose primary healthcare is not included in the joint municipal authority [in the region]. But we solved it quite simply, told them that you are with us in all the groups and meetings and then you apply [the guidelines] to your services. In practice, the joint municipal authority offered them the same support as others, even though they are not part of the organisation. We thought that we don’t [want to] separate some municipalities. (Interviewee 12)

Various manifestations of scarce resources often formed a trigger for initiating a change process, aligning with adapting instead of opting for absorbing (see Table 1). The need to change working principles in this manner arose from not having enough workforce, time, materials, money or technical solutions to perform effectively. The rotation of a health workforce was seen as a response to epidemiological and population service needs and, thus, interpreted here as an example of the initiation of an adaptive change process as a response to challenges arising from the changing operational environment. Finding resources for a vaccination programme was one of the biggest challenges mentioned by the interviewees. The need to ensure adequate skills and competencies as a form of resource among the workforce sometimes formed a hindrance to the adaptations previously mentioned.

The interviewees also contemplated the misjudgements they had made when leading responses at the beginning of the pandemic and the need to adapt to the consequences. This was illustrated by one of the leaders:

Well, in the beginning, we tried to keep a log and a written situational analysis of some kind. But yes, I have to say that, in a small municipality, the resources for this kind of thing that a memorandum would be made for everything are very scarce. After all, no official can leave things undone because he/she is afraid of some kind of misconduct. Surely someone can afterwards analyse our pandemic governance [in that situation], but there was no working model, so there was no model for how to act. And we did as we did in that situation. Probably something went wrong, or we exceeded our authority, but it was handled as it was handled. (Interviewee 22)

This exemplifies how adaptation was also represented in the data about an organisation’s or a local health system’s internal and constantly evolving contextual challenges of knowledge-forming instead of only to new external triggers.

### Transforming: expanding organisational boundaries and service configuration

Diversifying the selection of professionals in service delivery outside the scope of health and social care professionals was the most frequently mentioned example, which could be interpreted as a transformation in our data (Table 1). The initiation of this line of a change process in thinking and reframing the workforce was described by one interviewee:

And it has kind of opened eyes there, but also probably on the health and social services side, that we don’t limit ourselves to that group of health and social service professionals but that we should also look a little more broadly, in different crisis situations, to what resources the society has available. (Interviewee 1)

The initiation of transformative change processes in service delivery and the workforce was triggered by the necessity of understanding and reacting comprehensively to different needs arising from the COVID-19 crisis that could not be managed by health services alone. In addition to the triggers related to sensemaking, the mere availability of other types of workforces led to innovative activities in which these professionals could benefit a population’s well-being. Leaders described the initiation of change processes that could be interpreted as transforming to occur in service delivery when the availability of new professional groups due to shutdowns of other (municipal) services, such as libraries and city orchestras, effectively triggered the generation of new services. Other than health professionals were recruited to contact an elderly population to ensure their well-being, and new collaborations with commerce were launched. These examples show how limited resources can function as triggers for transformation.

The same kind of widening of working principles and thinking referred to in the heading of this subsection as “expanding boundaries” within an organisation was brought up by the leaders. This more comprehensive understanding of other sectors’ principles and operation modes seemed to be one of the main lessons the leaders wished to preserve, even at normal times. The interviewees widely hoped that advances in knowledge-based management and digital solutions in service delivery and other health and social service functions would also be maintained, as narrated by one interviewee:

We have also learned. For example, the digitalisation of the COVID-19 sampling process would never have been successful without this crisis. The way it was, the organisations that couldn’t work together, three different bodies had to come to an agreement that couldn’t have been reached in “peacetime” because there were disagreements on matters of principle, but now that there was a common crisis, we were able to overcome them. (Interviewee 17)

Transforming was manifested in the data particularly by framing the workforce in novel ways. Factors that hindered a transformation were not explicitly described by the interviewed leaders. Some can be identified by analysing triggers that lead to other responses (absorbing, adapting and resisting), such as existing structures and processes. Transforming was triggered particularly by a changing operational environment, the social dimension (e.g. population well-being) and new resources.

### Resisting: on good grounds or non-reflexively, with mixed effects

In the data, triggers that brought about resisting change were issues of conflicting values among different governance levels. For instance, one value conflict concerned attitudes towards the workforce, where some of the leaders called for a more humane approach to human resource issues in discussions about using legislation to force healthcare professionals to work. Another example of a contextual challenge that triggered resistance to change mirrors the social dimension of triggers and hindrances in descriptions of how local-level leaders consider the overall well-being of a population, especially those in vulnerable positions, with a broad mindset.

These leaders resisted strict restrictions, such as school closures or rundowns of health and social services, as proposed by national authorities:

But then, at some point, we closed the schools at the Ministry’s request. We closed them, although we might not have wanted to close them just yet. However, the pandemic situation is quite serious right now, so it is understandable that the schools are now closed. (Interviewee 3)

Interviewees reported incidents in which support from other people, often in higher positions, was or was not present. Both the support and lack of it could, depending on the context, trigger or hinder resistance to action. At the beginning of the pandemic, this was experienced when peers and superiors in an organisation still lacked an understanding of an upcoming crisis and were presented as interpretations of normalcy. These examples fall into the social dimension of triggers and hindrances. In contrast, the sheer impossibility of acting according to higher authorities’ demands was represented in the data as resistance to change. It could be triggered by contextual challenges, such as scarce material or human resources or discrepancies with legislation, such as not having the power to act in a certain way:

The question was whether we did or did not do what the minister wanted us to do. Well, if she had no jurisdiction over that question, then we came up with our own plan. (Interviewee 7)

Resisting could be characterised as “automatic” (i.e. non-reflexive) if the need for absorbing, adapting or transforming approaches was not identified. Descriptions of less reflection were present, especially concerning situations with time constraints, as the operational environment changed quickly. Also, the uncertainty of a situation could direct decision-making to preserve usual functions. Again, in the data, there were a few examples of hindrances to resisting change. However, the above-mentioned rapid changes could create considerable pressure to act and, consequently, sometimes even useful resistance could be hindered. The need to build an understanding of the pandemic situation seemed to act as a trigger to set back decision-making (i.e. resisting), whereas the same need in another context could trigger activities that can be classified as absorbing, adapting or even transforming.

An example of delayed decision-making was recounted by an interviewee:

And then we took care of it after we had been wondering for two days. Then, in the joint municipal authority, we took on the role of thinking about how to get the same management model to the whole province quickly. (Interviewee 12)

Thus, resisting as an approach to change was mainly characterised by two different groups of triggers. First, value conflicts represented the conscious reasoning behind the resistance. Second, the more automatic (i.e. non-reflexive) side of resisting was grounded in disregarding the need for changing organisational functions and believing that current operations were good enough to address the challenges.

## Discussion

This study investigated how local-level leaders’ approaches to operational changes during the COVID-19 crisis reflect resilience capacities and what triggers and hinders the initiation of these changes in a local health system context. This study responded to the lack of research on localised trust and the importance of including different health system levels in the field of resilience research (Saulnier *et al.*, 2023). By concentrating on the local health system level, we produced additional knowledge to previous research on the role of leaders in building resilience, adaptability and collaboration (Karreinen *et al.*, 2023a; Kihlström *et al.*, 2022; Ree *et al.*, 2021).

From a theoretical perspective, our aim was to enrich the understanding of the interconnections between different levels of health system resilience. Accordingly, we used health system resilience capacities as a theoretical framework to investigate local-level responses to a shock. Using this approach, we aimed to bridge local-level data with research conducted at the health system level. Second, we analysed context-specific events that either triggered or hindered the initiation of local-level operational changes and found that some of these events originated at other system levels. Consequently, our findings underline the entwinement of resilience at different system levels and the importance of studying these interdependencies (Tan *et al.*, 2023).

Third, the results indicate that leaders’ approaches to change potentially play a crucial role in the initiation of organisational responses. We suggest that this can have wide-ranging effects on the health system resilience as a whole. Furthermore, it is crucial that promoting awareness about leaders’ approaches to change and resilience capacities be incorporated into leadership training and preparedness planning. In the following sections, we discuss these theoretical and practical implications in more detail, including a critical reflection on the limitations of this research and concluding remarks.

### Theoretical implications

The descriptions by the interviewed leaders portray well how their approaches to change were triggered or hindered by various context-dependent factors, such as conflicting interests, differing understanding of the situation or availability of workforce and materials. The results also depict how leaders’ approaches to change may shape organisational and, at times, even local system-level responses during a major shock. By summarising definitions (Table 1) and testing these definitions in empirical analyses of local-level data, this study promotes the operationalisation of health system resilience capacities (i.e. concepts of absorbing, adapting and transforming) at the local health system level.

Overall, research on health system resilience has increasingly emphasised the importance of acknowledging its’ process-like qualities (Biddle *et al.*, 2020; Witter *et al.*, 2023). Our research complements these studies by viewing resilience as a process and by focusing our analyses at the initiation stage of change processes that potentially advance resilience capacities.

Resisting change, which was added to our analytical framework during the abductive analysis process (see “Resisting” in Table 1), has not been widely discussed in the health system resilience literature. Foroughi *et al.* (2022) referred to resistance as robustness, coping and stability, allowing for maintenance of system characteristics and continuity of operations. To complement these notions, our findings illustrate how resisting change can potentially strengthen resilience by taking time for collaboration, sensemaking and more reflexive decision-making. In previous studies (Karreinen *et al.*, 2023a; Lyng *et al.*, 2022), these have been found to support a more holistic view of crisis governance.

In this current study, a resisting mindset was partly portrayed as revocable and led to a more profound evaluation of the information at hand (and its insufficiency). Future research can explore, for example, the role of non-adaptive spaces in health system resilience. However, resistance can also have detrimental effects at different system levels, leading to the inability to adapt to and transform when circumstances require. These findings underline the importance of simultaneously considering responses at different system levels and ensuring that the diverse characteristics of health systems as complex adaptive systems are contemplated (Barasa *et al.*, 2018; Ignatowicz *et al.*, 2023; Witter *et al.*, 2023).

Our study shows that organisations readily took actions that can be classified as adapting or even transforming without first going through the absorbing phase. Hence, our analysis demontstrates that from a local-level perspective, resilience capacities (absorbing, adapting and transforming) do not necessarily follow each other linearly, as opposed to what can be understood from some previous system-level conceptualisations (e.g. Tan *et al.*, 2023; Thomas *et al.*, 2020). As a contribution to theoretical discussions about health system resilience, it can be deduced that absorption, adaptation, transformation and resistance are not prerequisites for each other.

A significant conclusion from the perspective of leadership skills is that a shift from one way of responding to another is not necessarily a conscious procedure. The analyses revealed that different resilience capacities were present in the organisations coincidently. For example, a transforming mindset could prevail concerning workforce issues, while, at the same time, management was engaged with an absorbing approach. This interplay and non-linearity of different approaches deserves to be studied in detail, and it should also be more fully acknowledged in the conceptualisations of health system resilience.

Our findings further illustrated how the context in which a challenge occurred influenced whether it functioned as a trigger or as a hindrance. This emphasis on contextuality is well in line with previous research highlighting the importance of understanding resilience as a highly context-dependent phenomenon (Duchek, 2020; Ignatowicz *et al.*, 2023; Witter *et al.*, 2023) as well as studies exploring the complexities of organisational structures and their changes (Watson *et al.*, 2024). We propose that a context should be understood broadly as including operational and local environments, health system levels, the type of shock or stress facing a system as well as changes that happen at other system levels or in society (see also, Ignatowicz *et al.*, 2023).

Excerpts on resisting and transforming as approaches to change were outnumbered by those exemplifying absorption and adaptation. This may have been partly caused by the timing and the contents of the interviews, as, according to the literature, absorbing and adapting are identified as the reactions mostly seen in the first stages of a shock (Thomas *et al.*, 2020). Our finding on the limited number of examples of transforming echoes that of Saulnier *et al.* (2023). By understanding transformation as sometimes a minor-scale change instead of a system-wide perspective and permanent solution, it becomes more reasonable in a local health system context. For instance, using someone other than health professionals to deliver part of health services was a temporary arrangement. Nonetheless, this exemplifies a novel way of thinking in a local health system context. The questions that remain unanswered include whether making profound transformative choices is less convenient, ineffective or just impossible during a crisis when chaotic circumstances may prevail, or how enough space and time for reflexive thinking can be ensured. We suggest that the meaningfulness of transformation is not weighed by the extent of a change, and even minor shifts in reasoning can be regarded as fundamental.

While most health system resilience research is carried out at the national health system level, we focused on local health systems. The role of the local level was especially significant during the COVID-19 pandemic in the Finnish healthcare system as it was highly decentralised at the time of our data gathering. Resilience frameworks and theories that focus merely on single system components might not fully grasp these localised capacities and strengths that have a fundamental effect on the whole system’s resilience (Kihlström and Karreinen, 2024). This is why it is important to develop frameworks and conceptualisations that seek to enrich the health system resilience literature with an understanding of local-level governance and leadership.

### Practical implications

The variations in the occurrence of descriptions of different approaches to change might also reflect the differences in the leaders’ roles in directing the organisational response. As stated above, absorbing and adapting are largely guided by prior planning. Instead, resisting and transforming require leaders to take a stronger stance towards challenges. The latter two also entail a closer consideration of values. An inference can be thus made that resisting and transforming depend more heavily on the leader’s disposition towards change, which raises another aspect for future research, entailing the use of power (Duchek, 2020; Kihlström *et al.*, 2023; Topp, 2020; Witter *et al.*, 2023). Evaluating the implications of absorbing (and adapting) possibly happening without the leaders’ awareness and how this affects the system’s resilience is important. Additionally, it is noteworthy that transformative thinking amid a crisis was represented in the data as something that leaders appreciated, suggesting that this theme is important and should be covered in crisis management training.

As a further practical implication, our results highlight the need to include reflexivity in both decision-making and leadership training. Decisions at different levels should be made being aware that the context-bound factors and the leaders’ approaches to change influence the chosen responses. To promote legitimacy and openness as well as the quality of preparedness planning, we suggest that leaders are trained and equipped to contemplate on the multifaceted sides of crises, responses and resilience before, during and after shocks that they encounter. This consideration could be facilitated, for instance, by exploiting resilience frameworks that consider both whole-system perspectives and contextual and local-level challenges. Examples of such supporting practices include using the health system resilience testing tool, which is based on the assessment of the four health system functions (Zimmermann *et al.*, 2024) or asking the four core questions of resilience, namely, resilience for what, to what, of what, and through what (Wiig *et al.*, 2020).

Overall, there is a widespread consensus that effective leadership plays a key role in building health system resilience (Ellis *et al.*, 2023; Saulnier *et al.*, 2023; Tan *et al.*, 2023; Thomas *et al.*, 2020). However, according to our data, a leader’s favourable approach to change is insufficient, as the surroundings can induce hindrances that may hamper the leader’s efforts. For instance, scarce resources of materials or workforce can force an organisation to transform services, or scarcity can hinder some actions if they are impossible to fulfil, thus triggering resistance. Also, support or lack of it from other actors to the leaders, be it from higher-level authorities or clinical professionals, reasonably affects the initiation of a change process in an organisation. These complex interrelationships between health system levels and the contextuality of triggers and hindrances to change should be acknowledged not only theoretically in health system resilience frameworks but also in practical leadership training for crisis management.

### Limitations

The focus of this empirical qualitative study was on the initiation of responses to a shock (i.e. change processes) in local health systems. While this study focused on the events during a pandemic, it can inform research and management of other forms of changes and shocks, such as climate change. The evaluation of the reached levels of resilience is outside the scope of this study. The data do not enable us to evaluate or measure how these change processes have developed or affected the overall resilience of an organisation or health system. Such longitudinal as well as multinational study designs are important for further research because these research designs can bring new knowledge about health system resilience, recovery and learning.

The methodology of this study can partly explain some of our findings. First, the emphasis on examples of adapting and absorbing might be due to the interviewers asking specifically about how service delivery was adapted during the COVID-19 pandemic. Second, the timing of the interviews (i.e. one year from the beginning of the pandemic) can accentuate the actions performed rapidly and perhaps with less reflection. Third, interviews as a data-gathering method may underestimate the number of less reflexive actions (such as absorbing and partly resisting), as answering interview questions itself requires contemplation.

The study sample, as is common in qualitative research, is small. Therefore, this study does not aim to generalise the findings to other health systems. However, our detailed description of the analysis process adds to the transparency of this current study and allows other researchers to design similar studies or further develop their research based on the theoretical, conceptual and practical contributions of this paper. While qualitative studies cannot be repeated exactly in the same form, nor can results be transferred to different contexts, this study offers theoretical and practical notions for further studies exploring the concepts of absorbing, adapting, transforming and resisting in connection to health system resilience.

## Conclusions

This study provides empirical evidence of how health system resilience centres around processes instead of outcomes. Resilience is closely connected to different forms of change and also resistance to change. For a long time, leadership has been acknowledged as crucial in promoting resilience. This study draws attention to the initiation of change processes at the upper and middle management levels in the context of local health systems. Here, leaders’ approaches to change play a pivotal role as decisions on responses are made. Our results suggest that leaders’ approaches to change reflect health system resilience capacities and may affect organisational and local level responses and thereby health system resilience. To ensure that the course of events is evolving in a favourable direction from the perspective of health system resilience, awareness and reflection of leaders’ standpoints and approaches to change are needed. Allowing multiple voices and shared sensemaking can facilitate weighing and assessing decisions.

We provide evidence that the resilience capacities (absorbing, adapting, transforming or resisting) are employed in a non-linear manner and can co-exist. The combinations of the health system resilience capacities are evolving and changing throughout the crisis. It is also important to recognise that none of the capacities are self-evidently “good” or “bad” approaches to health system resilience. Depending on the circumstances, all of these factors may either promote or impede resilience.

The starting point of a change process is crucial and the factors that trigger or hinder the initiation of this process should be identified. This study revealed that various triggers and hindrances to initiate a change process can lead to varying responses depending on the context and a leader’s approach to change. The research results underline the need to address how processes at different health system levels are intertwined and connected to health system resilience as a whole. This is important both in research and in the governance of health systems.
